# Isoaspartate and neurodegeneration

**DOI:** 10.18632/aging.204420

**Published:** 2022-11-29

**Authors:** Jijing Wang, Sourav Mukherjee, Roman A. Zubarev

**Affiliations:** 1 Department of Medical Biochemistry and Biophysics, Karolinska Institutet, Stockholm, Sweden

**Keywords:** neurodegeneration, deamidation, isoaspartate

Though the etiology of Alzheimer’s disease (AD) and many other neurodegenerative diseases are still unclear, age is known to be the biggest risk factor for neurodegeneration. And as most neurodegenerative diseases are proteopathies, protein aging is likely to be involved in the disease mechanism.

One of the major products of protein aging is isoaspartate (isoAsp), a damaging amino acid residue generated either from deamidated asparaginyl (Asn) or, less frequently, isomerized aspartatyl (Asp) residue. Both deamidation and isomerization processes occur spontaneously at physiological conditions and require no enzymes. In isoAsp, the rearrangement of a CH_2_ group from the side chain extends the polypeptide backbone, disrupting the protein native structure and affecting its function. Deamidated proteins are prone to aggregation due to the loss of native conformation. In 1991 it was found that isoAsp formation in amyloid beta (Aß) peptide facilitates its aggregation [1], possibly triggering AD, but the isoAsp hypothesis of AD has been slow to provide supporting evidence. Two decades later we have uncovered a significant association of elevated isoAsp levels in blood proteins with AD [2]. In subsequent proteomic study, we demonstrated strong relationship between the easily aggregated proteins (e.g., fibrin and fibrinogen) in blood with rapid AD development [3]. These proteins tend to interact with Aβ, facilitating Aβ fibrillization and formation of fibrin clots resistant to degradation.

Nature has foreseen a process of isoAsp repair, albeit not to the original Asn but to a healthy _L_-Asp. Using S-adenosylmethionine (SAM) as a methyl donor, the enzyme protein _L_-isoaspartyl methyltransferase (PIMT) methylates isoAsp, which upon the spontaneous loss of methanol becomes Asp (< 25% of cases), or, more often, isoAsp again [4]. With age SAM production declines, rendering even such low-efficiency repair insufficient. This results in isoAsp accumulation in long-lived proteins, such as human serum albumin (HSA). HSA is the most abundant protein in blood, with circulation half-life time of three weeks.

To test the role of isoAsp in neurodegeneration, one needed to quantify the isoAsp levels in blood proteins of larger cohorts, which was not a trivial task due to the absence of commercial anti-isoAsp antibodies. While isoAsp is slightly immunogenic, the small chemical difference between isoAsp and Asp renders production of antibodies against isoAsp difficult. After several unsuccessful attempts we managed to develop a monoclonal antibody (mAb) with high sensitivity and specificity against isoAsp in an important domain of HSA [5]. Using indirect ELISA and artificially deamidated HSA for its calibration, we established for the first time the normal isoAsp range in HSA, (0.74 ± 0.13)%, by analyzing blood in 100 healthy donors [5]. In a subsequent recent publication [6], we demonstrated that deamidation-induced aggregation diminishes the binding capacity of HSA toward Aβ peptide and phosphorylated tau (p-Tau) protein. The size exclusion chromatography proved the increased level of HSA aggregates in patients with AD compared with controls. By the developed ELISA, we found a significant increase of isoAsp level in HSA of the AD blood compared with healthy controls, as well as elevated levels of free Aß not bound with HSA. More intriguingly, we discovered in the same AD group a significantly reduction of endogenous antibodies against deamidated HSA. Based on these findings, we updated the isoAsp hypothesis of AD etiology, highlighting the role of isoAsp accumulation in HSA and reduction of anti-isoAsp mechanism as AD risk factors that contribute to diminished clearance of Aß and pTau.

Subsequently, via another cohort composed of a different ethnic group (Asian versus predominantly European), we validated the previous results. We also discovered the potential of isoAsp-related biomarkers in diagnostics of other neurodegenerative diseases, including frontotemporal dementia, vascular dementia and, to lesser extent, Parkinson’s disease. The most significant discovery was the best performance of isoAsp-related biomarkers (AUC = 0.92) in detection of mild cognitive impairment (MCI) compared with other current blood biomarkers (Aβ42/Aβ40 ratio, p-Tau181, neurofilament light chain (NfL) and glial fibrillary acidic protein (GFAP)). In addition, the levels of isoAsp in HSA and its antibodies were found to strongly correlate with cognitive decline, which strengthened the role of isoAsp in neurodegeneration.

Being intrigued by the apparently irreversible nature of deamidation that seems to be in stark contrast with observed cell self-renewal phenomena (e.g., totally normal “young” babies born by woman 60 and more years old), we investigated the possibility of true isoAsp-to-Asn repair. Besides reviewing the available theoretical arguments, we reported the first experimental evidence on the reversibility of isoAsp via protein succinimide/isoaspartate ammonia ligase (PSIAL) activity [7]. Such activity was found in e.g. human cytoplasmic aspartate aminotransferase, but other proteins are likely to possess PSIAL activity as well.

Collectively, the accumulated results provide new venues for early diagnostics, therapy and perhaps even preventions of neurodegeneration and other age-related diseases.
